# Improvement of renal oxidative stress markers after ozone administration in diabetic nephropathy in rats

**DOI:** 10.1186/1758-5996-2-29

**Published:** 2010-05-13

**Authors:** Mohamed D Morsy, Waleed N Hassan, Sherif I Zalat

**Affiliations:** 1 Department of Physiology, College of Medicine, Menoufiya University, Egypt; 2 Department of Biochemistry, College of Medicine, Menoufiya University, Egypt; 3 Department of Anesthesia, College of Medicine, Menoufiya University, Egypt

## Abstract

**Background:**

Several complications of diabetes mellitus (DM) e.g. nephropathy (DN) have been linked to oxidative stress. Ozone, by means of oxidative preconditioning, may exert its protective effects on DN.

**Aim:**

The aim of the present work is to study the possible role of ozone therapy in ameliorating oxidative stress and inducing renal antioxidant defence in streptozotocin (STZ)-induced diabetic rats.

**Methods:**

Six groups (n = 10) of male Sprague Dawley rats were used as follows: Group C: Control group. Group O: Ozone group, in which animals received ozone intraperitoneally (i.p.) (1.1 mg/kg). Group D: Diabetic group, in which DM was induced by single i.p. injections of streptozotocin (STZ). Group DI: Similar to group D but animals also received subcutaneous (SC) insulin (0.75 IU/100 gm BW.). Group DO: In which diabetic rats received the same dose of ozone, 48 h after induction of diabetes. Group DIO, in which diabetic rats received the same doses of insulin and ozone, respectively. All animals received daily treatment for six weeks. At the end of the study period (6 weeks), blood pressure, blood glycosylated hemoglobin (HbA_1c_), serum creatinine, blood urea nitrogen (BUN), kidney tissue levels of superoxide dismutase (SOD), catalase (CAT), glutathione peroxide (GPx), aldose reductase (AR) activities and malondialdehyde (MDA) concentration were measured.

**Results:**

Induction of DM in rats significantly elevated blood pressure, HbA_1c_, BUN, creatinine and renal tissue levels of MDA and AR while significantly reducing SOD, CAT and GPx activities. Either Insulin or ozone therapy significantly reversed the effects of DM on all parameters; in combination (DIO group), they caused significant improvements in all parameters in comparison to each alone.

**Conclusions:**

Ozone administration in conjunction with insulin in DM rats reduces oxidative stress markers and improves renal antioxidant enzyme activity which highlights its potential uses in the regimen for treatment of diabetic patients.

## Background

A high percentage of type I and II diabetic patients eventually develop diabetic nephropathy (DN) that may progress to end-stage renal disease (ESRD) [1]. Type I diabetes mellitus (DM) usually manifests early in life. So, ESRD may develop at a relatively younger age, producing an additional burden for patients and health services [2]. The structural injury in DN develops before the clinical and laboratory abnormalities such as hypertension, albuminuria or reduction of the glomerular filtration rate [3,4]. Thus, waiting for clinical or laboratory manifestations of renal disease before starting treatment may hinder efforts to prevent ESRD. Several pathways involving hemodynamic and metabolic factors have been implicated in the pathogenesis of DN including oxidative stress [5], activation of protein kinase C [6], increased formation of advanced glycation end products [7] and polyol-hexosamine pathway flux [8]. The excessive production of reactive oxygen species (ROS) has been suggested as a common outcome from all these pathways leading to increased oxidative damage at the level of lipid peroxidation [9] and culminating in DN in association with DM [10]. Thus, any treatment which is able to stabilize oxygen metabolism and modulate oxidative stress would help to delay the onset of DN in diabetes mellitus.

Many controlled trials have examined the validity of using ozone as a therapeutic agent for the treatment of several disorders [11]. Ozone therapy stimulates the antioxidant response in cardiomyopathy patients [12] and increase oxygen unloading capacity of hemoglobin in diabetic patients [13]. Ozone administration also been shown to exert a protective effect against liver damage induced by carbon tetrachloride and renal ischemic-reperfusion injury by an oxidative preconditioning mechanism that stimulates antioxidant endogenous systems and modulates nitric oxide (NO) production [14]. The main purpose of this work is to determine the role of ozone administration in ameliorating oxidative stress in STZ-induced DN in diabetic rats so as to establish its potential use in the strategy for the treatment of diabetic patients.

## Methods

### Experimental animals and groups

This study was carried out on 60 male Sprague-Dawley rats weighing between 150 and 200 g obtained from the National Research Centre, Cairo, Egypt. The animals were divided into six equal groups. Animals were fed with a standard laboratory chow and water, ad libitum, and housed in the animal house of Menoufiya Faculty of Medicine, Egypt with a 12:12-h light/dark cycle. The study groups were as follows: **Group C**: Control group, injected (i.p.) with citrate buffer (0.1 M, pH 4.5). **Group O**: Ozone group, given ozone treatment i.p. at a dose of 1.1 mg/kg. This dose of ozone has been shown to achieve oxidative preconditioning without appreciable toxicity [15]. **Group D**: Untreated diabetic group. DM was induced by single i.p. injections of streptozotocin (STZ). **Group DI**: Rats made diabetic as in group D were given mixtard insulin 30 (40 IU/ml) subcutaneous (Novo Nordisk, Denmark) at a dose of 0.75 IU/100 gm BW once daily in 0.75 ml volumes [16]. **Group DO**: Diabetic rats received the same dose of ozone as group O, 48 h after induction of diabetes. **Group DIO**: Diabetic rats received the same doses of insulin and ozone as in groups DI and DO, respectively. Groups C, D and DO also received SC injections of 0.75 ml saline daily, while groups C, D and DI were injected i.p. with oxygen (the vehicle for O_3_) in the same gas volume as the ozone/oxygen mixture. All animals were treated daily for six weeks.

### Induction of diabetes and ozone preparation

DM was induced with single IP injections of STZ (45 mg/kg) in freshly prepared citrate buffer (0.1 M, pH 4.5) (Sigma Chemical Company, USA), while control rats were injected with vehicle buffer only. DM was verified by measuring blood glucose in tail nick blood samples. Rats with non fasting blood glucose levels of ≥20 mmol/L after 48 h of STZ injection were considered diabetic [17]. The study duration was 6 weeks, a period which has been shown to induce detectable diabetic complications in the kidney [18,19]. Ozone was generated with ozonator equipment (Ozone Longevity Resources Dwyermade, Canada). The ozone concentration in the O_3_/O_2 _mixture was 50 μg/ml and was i.p. administered. Ozone was obtained from medical grade oxygen and was used immediately upon generation and represented only about 3% of the O_3_/O_2 _gas mixture. The ozone concentration was measured using a UV spectrophotometer at 254 nm. The ozone given to each animal was adjusted to a final dose of 1.1 mg/kg BW [20].

### Measurement of blood pressure

At the end of the experimental period, blood pressure was measured using the rat-tail sphygmomanometer technique (Harvard Apparatus Ltd., England) [21]. Systolic blood pressure (SBP) and mean arterial blood pressure were estimated from recorded graphs. Diastolic blood pressure (DBP) was calculated from the expression - Mean pressure equals diastolic pressure plus one third pulse pressure [22].

### Blood and tissue sampling

At the end of the 6th week, retro-orbital blood samples were obtained under anaesthesia (sodium thiopental at 40 mg/kg IP Bio-chem, Austria) [23]. The samples were divided into two parts. The first part was allowed to clot for 20 min, and then centrifuged at 14,000 rpm for 10 min to obtain serum used for the determination of BUN and creatinine levels. The other part was collected in tubes containing potassium oxalate and sodium fluoride and used to measure plasma glucose and HbA_1c_. The animals were killed by decapitation; kidneys were dissected out, cut into small pieces and homogenized in ice-cold 50 mM Tris, 1.0 mM EDTA, pH 8.0, with 10 mM 4-(2 aminoethyl) benzenesulfonyl fluoride (AEBSF), 2 mM dithiothreitol, 5 mM leupeptin, 2 mM pepstatin, using an Omni tissue homogenizer (Omni international, Gainesville, VA). The extracts were centrifuged at 14,000 rpm for 30 min at 4°C. The supernatant was stored at -80°C for various assays.

### Biochemical analyses

Serum BUN was determined by an enzymatic colorimetric method using a commercial kit (Boehringer Manheim, Germany). This method is based on urease (urea amidohydrolase)/glutamate dehydrogenase coupled reactions and uses a two-point fixed-time kinetic scheme for monitoring the rate of consumption of NADH at 340 nm [24]. Serum creatinine was determined by the Jaffe reaction in which creatinine reacts with picrate ion in an alkaline medium to yield an orange red complex which was measured at 490 nm [25]. Plasma glucose was determined using the glucose oxidase method [26]. Addition of peroxidase and a chromogenic oxygen acceptor results in the formation of a coloured compound that can be measured at 500 nm (Life Scan, CA, USA). HbA_1c _was determined according to the chemical separation and colorimetric method based on the phenol sulphuric acid reaction of carbohydrates. Hemolyzates were treated with 1 mol/l oxalic acid in 2 mol/l HCl for 4 h at 100°C; the protein was precipitated with trichloroacetic acid, and the free sugars and hydroxymethyl furfural in the supernatant were treated with phenol and sulphuric acid to form the colour [27]. MDA concentration was measured in tissue homogenates after precipitation of protein with trichloroacetic acid. Thiobarbituric acid (TBA) reacts with MDA to form TBA reactive product, which was measured at 532 nm spectrophotometrically. An MDA solution freshly made by the hydrolysis of 1,1,3,3-tetramethoxy propane was used as standard [28]. The results were expressed as nmol of MDA per mg protein.

### Enzyme Activity Assays

**SOD (EC 1.15.1.1) activity **in kidney homogenates was assayed following the method developed by Nishikimi et al. [29] as modified by Kakkar et al. [30]. Five μg protein was mixed with sodium pyrophosphate buffer, phenazine methosulphate (PMT) and nitro blue tetrazolium (NBT). The reaction was started by the addition of NADH. The reaction mixture was then incubated for 90 seconds at 30°C and stopped by the addition of 1 ml of glacial acetic acid. The absorbance of the chromogen formed was measured at 560 nm. One unit of SOD activity was defined as the enzyme concentration required to inhibit chromogen production by 50% in one minute under the assay conditions. **CAT (EC 1.11.1.6) activity **was measured in homogenates by the method of Bonaventura et al. [31]. Five μg protein from the homogenate was mixed with 2 ml of 7.5 mM H_2_O_2 _and a time scan was performed for 10 min at 240 nm at 25°C. One unit of CAT activity was defined as the amount of enzyme decomposing 1 μmol of H_2_O_2 _per minute. **GPx (EC 1.11.1.9) activity **was assayed by Paglia and Valentine's method [32], using H_2_O_2 _and NADPH as substrates. The conversion of NADPH to NADP^+ ^was followed by recording the changes in absorption intensity at 340 nm (Ransel kit, Randox, UK), and one unit was expressed as 1 nM of NADPH consumed per minute/mg tissue. **AR activity **was determined in a reaction mixture containing sodium phosphate buffer (0.1 M, pH 6.2), 150 mM NADPH, 10 mM DL-glyceraldehyde, and the enzyme solution in a total volume of 1 ml. The reaction was started by the addition of enzyme and activity was measured by estimating NADPH oxidation as a decrease in absorbance at 340 nm. Assays were carried out at room temperature with appropriate blanks subtracted from each reaction to correct for nonspecific oxidation of NADPH during the measurement. One unit of enzyme activity was defined as the amount of enzyme catalyzing the oxidation of 1 mM of NADPH/min/mg tissue under the assay conditions [33].

### Statistical analysis

All data were expressed as means ± SEM. The means for the different groups were compared using Kruskal Wallis one-way ANOVA (one-way analysis of variance), followed by Post Hoc test. The level of significance for all comparisons was set at P < 0.05.

## Results

### Blood pressure measurements

There were significant increases in SBP and DBP of diabetic rats when compared to control group (C). However, treatment of diabetic rats with either insulin (group DI) or ozone (group DO) significantly reduced both SBP and DBP values when compared to group D (P < 0.01). Furthermore, combined treatment with insulin and ozone in group DIO significantly lowered both SBP and DBP blood pressure relative to D, DI and DO groups (P < 0.001) (Table 1).

**Table 1:** Effect of six weeks ozone treatment on different parameters in STZ induced diabetes in rats.

Group	SBP (mmHg)	DBP (mmHg)	**(Hb A**_**1c **_**%)**	BUN (mmol/L)	Creatinine (μmol/L)
C	106.1 ± 5.9	88.3 ± 5.6	7.4 ± 1.36	5.72 ± 0.25	45.9 ± 2.6

O	101 ± 4.8	79.5 ± 2.1	7.1 ± 1.25	4.26 ± 0.82	36.2 ± 4.4

D	165.4 ± 6.7 * °	127.3 ± 4.2 * °	16.1 ± 1.47 * °	35.15 ± 4.35 * °	184.7 ± 1.8 * °

DI	109.5 ± 3.6 ° #	100.2 ± 4.1 ° #	8.3 ± 0.38 ° #	27.50 ± 5.79 * °	165.3 ± 11.4 * °

DO	102.5 ± 5.2 ° #	97.3 ± 5.3 ° #	8.9 ± 1.02 ° #	11.35 ± 5.37 * ° # ˆ	92.8 ± 15.1 * ° #

DOI	87.8 ± 6.5 # ˆ ¥	78.9 ± 3.2 # ˆ ¥	7.3 ± 0.24 # ˆ ¥	5.81 ± 0.73 # ˆ ¥	47.7 ± 0.88 # ˆ ¥

Effect of treatment with ozone on systolic (SBP), diastolic blood pressures (CBP), glycosylated Hb (Hb A_1c_), serum creatinine and blood urea nitrogen (BUN) (Means ± SE) in control and experimental groups of rats (n = 10).

* Significant change from the corresponding values in Non-diabetes control group.

° Significant change from the corresponding values in Non-diabetes ozone-control group.

# Significant change from the corresponding values in diabetes control group.

ˆ Significant change from the corresponding values in diabetes insulin treated group.

¥ Significant change from the corresponding values in diabetes ozone treated group.

### Glycosylated hemoglobin

HbA_1c _levels were significantly higher in diabetic animals when compared with controls (group C). Insulin treatment significantly reduced HbA_1c _in diabetic rats (group DI) relative to untreated diabetic animals (group D) (P < 0.001). Ozone treatment in diabetic rats (DO) reduced HbA_1c _significantly; while concomitant treatment with insulin and ozone in group DIO reduced HbA_1c _levels further, restoring them to near normal values (Table 1).

### Serum BUN and creatinine levels

Induction of diabetes in group D resulted in significant elevation of both BUN and creatinine when compared with control levels (C). Treatment of diabetic animals with insulin alone affected neither BUN nor creatinine significantly, while ozone administration alone significantly reduced both BUN and creatinine when compared to the diabetic group (D). Combined treatment with insulin and ozone in group DIO resulted in significant reductions of BUN and creatinine relative to either D or DI or DO groups, restoring them to near normal levels (Table 1).

### Kidney tissue SOD, CAT and GPx activities

**Figure 1:**
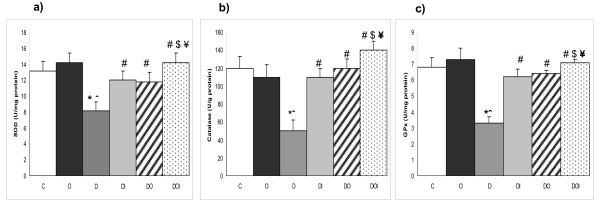
**The effects of ozone on renal SOD, GPx and CAT activities in diabetic nephropathy in rats**. The figure shows a) SOD, b) CAT, c) GPx activities in kidney tissues homogenate in the different studied groups (n = 10). C = control, O = ozone treated, D = diabetes mellitus, DI = diabetes+insulin, DO = diabetes+ozone, DOI = diabetes+ozone+insulin. (*) significant change from normal control group (p < 0.01). (ˆ) significant change from ozone control group (p < 0.01). (#) significant change from diabetic group (p < 0.01). ($) significant change from to diabetic insulin group (p < 0.01). (¥) significant change from diabetic insulin group (p < 0.01).

Whereas ozone therapy in control rats produced no significant changes in kidney tissue SOD, CAT and GPx activity levels, induction of diabetes in group D produced significant decreases in the activity levels of the three enzymes when compared with control rats (group C). Insulin and ozone treatment in groups DI and DO caused significant increases in kidney homogenate activity of SOD, CAT and GPx when compared with untreated diabetic rats (D). Concomitant administration of both insulin and ozone in diabetic rats produced significant increases in all measured antioxidant enzyme activities relative to their corresponding values in D, DI and DO groups, restoring them to near normal levels (Figure 1).

### Kidney tissue MDA concentration and AR activity

**Figure 2:**
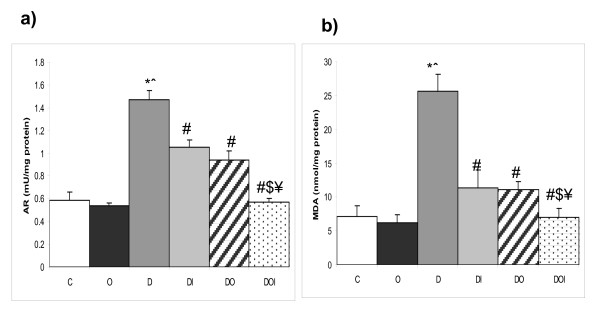
**The effects of ozone on renal MDA concentration and AR activity in diabetic nephropathy in rats**. The figure shows a) AR activity, b) MDA level in kidney tissues homogenate in the different studied groups (n = 10). C = control, O = ozone treated, D = diabetes mellitus, DI = diabetes+insulin, DO = diabetes+ozone, DOI = diabetes + ozone + insulin. (*) significant change from normal control group (p < 0.01). (ˆ) significant change from ozone control group (p < 0.01). (#) significant change from diabetic group (p < 0.01). ($) significant change from to diabetic insulin group (p < 0.01). (¥) significant change from diabetic insulin group (p < 0.01).

Induction of diabetes in group D significantly increased kidney tissue levels of MDA and AR enzyme activities when compared with the control group (C). Insulin treatment (DI) and ozone administration (DO) in diabetic rats independently produced significant decreases in both MDA and AR activity when compared with group (D). Concomitant administration of insulin and ozone brought MDA and AR activity levels to near normal levels by significantly greater reductions of activity levels relative to D, DI or DO groups (figure 2).

## Discussion

Oxidative stress in DM accelerates the development of diabetic micro- and macro-vascular complications through poorly understood mechanisms [34]. Hypertension, as a vascular complication of DM, is frequently seen in Type I DM and many factors are involved in its pathogenesis including the availability of nitric oxide, increased production of ROS and alterations in the renin-angiotensin system as well as dyslipidaemia [35]. There are evidences that, the formation of ROS is a direct consequence of hyperglycemia and is associated with the vascular complications seen in diabetic patients [2,36]. Induction of diabetes in the present study causes significant elevations of both SBP and DBP when compared to the control group. Either insulin (Group DI) or ozone (Group DO) treatment independently resulted in significant reductions in both SBP and DBP. This could be explained by the vasodilator effect of both agents with their ability to induce endothelial cell NO production [14,37].

Development of DN in the present study was confirmed by significant elevations of serum creatinine and BUN as well as the development of hypertension, as earlier reported by Knoll et al. [18]. The significant reduction of renal antioxidant enzyme activities with significant elevation of MDA and AR may be related to a higher level of oxidative stress in diabetic rats. Oxidative stress may be responsible for several complications of DM, including nephropathy, retinopathy and atherosclerotic vascular diseases due to overproduction of ROS with subsequent lipid peroxidation [38,39]. Hyperglycaemia reduces the synthesis and activities of a number of antioxidant enzymes including SOD and GPx, presumably by glycation [5]. It is well established that diabetic patients have a lower antioxidant defences, enzymatic (SOD, CAT, GPx) and non-enzymatic (vitamin C, E or A, free radical scavengers) [5]. The role of oxidative stress in the pathogenesis of DN is not only through overproduction of ROS but also through the reduction of antioxidant enzyme activities, auto-oxidation of glucose, impaired glutathione metabolism, formation of lipid peroxides and non-enzymatic protein glycosylation [40,41]. Oxidative stress may also induce the formation of angiotensin converting enzyme, protein kinase C and mitogen-activated protein kinase (MAPK) activation that help in the aggravation of DN in diabetic patients [42].

The efficacy of ozone therapy in DM has been attributed to its hypoglycemic effect, induction of antioxidant enzyme activities [33,43] and control of their expression [20,44]. The significant reduction of HbA_1c_, kidney tissue MDA and AR levels with significant elevation of kidney tissue SOD, CAT and GPx activities by both ozone and insulin treatment may be explained by their hypoglycemic effect with subsequent reduction of metabolic complications. This view is supported by Rodriguez et al. [12] who found that controlling hyperglycemia in diabetic patients with insulin or other hypoglycemic agent reduces oxidative stress-induced DN complication in diabetes patients. Another possible mechanism could be through ozone-induced NO synthase expression at the level of the renal vascular endothelium resulting in arteriolar vasodilatation which prevents the development of DN [45]. Furthermore, ozone has been shown to enhance antioxidant endogenous systems by means of oxidative preconditioning and adaptive mechanisms which reduce vascular complications in diabetic patients [46]. These findings support our hypothesis that ozone has a renal protective role against oxidative damage in diabetes mellitus.

The antioxidant-prooxidant balance, associated with the regulatory effects of ozone on AR activity represents another interesting action of this complementary therapeutic approach. Aldose reductase is a key enzyme in the polyol pathway and its inhibitors have been used as therapeutic drugs linked to the enhancement of NO production or release [47]. Ozone may also protect against the imbalance in NO/ROS by preventing NO depletion and improving NO-mediated vasodilatation in diabetic rats to restore blood pressure to normal values [43]. Another explanation is that ozone treatment decreases the overproduction of ROS by the mitochondrial electron transport chain in diabetics, thus inhibiting the activation of the polyol pathway and the increasing in the concentration of angiotesin enzyme activity [48]. Finally, Ozone could also induce SOD activity through stimulation of the SOD gene expression [47].

Both insulin and ozone may act synergistically to reduce HbA_1c _level, blood pressure and ROS production while stimulating antioxidant enzymatic activities to the control levels. Both of them independently exert hypoglycemic effects [33,43]. In addition, ozone enhances antioxidant enzyme activity and reduces overproduction of ROS which together act to protect cell membranes [12]. Also ozone treatment partially reduce the imbalance between the generation of ROS and scavenging enzyme activity [49], while insulin may stimulate glucose transporters gene expression at the cellular level to control the hyperglycemic complications of diabetes [50]. Also, insulin may correct the deleterious effects of hyperglycemia at the cellular level of the GPx by increasing the level of nuclear factor kappa B [51]. Thus both insulin and ozone administration protect diabetic animals from the deterioration of DN.

## Conclusions

This study explored the possible mechanisms by which ozone may improve oxidative stress levels and renal antioxidant system in experimental diabetic rats. So ozone therapy may be considered as an adjuvant to insulin in the treatment of diabetes to prevent or alleviate diabetes induced nephropathy. This opens the way for long-term studies to confirm the beneficial effects of ozone administration in diabetic animal models.

## Competing interests

The authors declare that they have no competing interests.

## Authors' contributions

MMD participated in the design of the study, performing of the experiments and helping draft the manuscript. HWN participated in performing the experiment and revised the manuscript especially figures and tables. ZSI prepared the ozone doses freshly and participated in revision of the manuscript. All authors have read and approved the manuscript.

## References

[B1] Greald B Appel MD Preventing or slowing the progression of diabetic nephropathy BUMC Proceedings 1999 12 6

[B2] Heidland A Bahner U Deetjen A Götz R Heidbreder E Schäfer R Teschner M Mass-screening for early detection of renal disease - benefits and limitations of self-testing for proteinuria J Nephrol 2009 22 2 249 54 19384843

[B3] Mojahedi MJ Bonakdaran S Hami M Sheikhian MR Shakeri MT Aiatollahi H Elevated serum C-reactive protein level and microalbuminuria in patients with type 2 diabetes mellitus Iran J Kidney Dis 2009 3 1 12 6 19377253

[B4] Caramori ML Kim Y Huang C Fish AJ Rich SS Miller ME Russel G Mauer M Cellular basis of diabetic nephropathy: Study design and renal structural- functional relationships in patients with long-standing type 1 diabetes Diabetes 2002 51 506 13 10.2337/diabetes.51.2.506 11812762

[B5] Yoshida SI Hashimoto T Kihara M Imai N Nomura K Hirawa N Toya Y Kitamura H Umemura S Urinary oidative stress markers closely reflect the efficacy of Candesartan treatment for diabetic nephropathy Nephron Exp Nephrol 2008 111 20 30 10.1159/000178764 19052474

[B6] Ibrahim S Rashed L Fadda S Evaluation of renal gene expression of protein kinase C isoforms in diabetic and nondiabetic proliferative glomerular diseases Scientific World J 2008 31 835 44 10.1100/tsw.2008.108 PMC5849071 18758661

[B7] Miyata T Dan T Inhibition of advanced glycation end products. an implicit goal for the treatment of diabetic nephropathy Diabetes Res Clin Pract 2008 13 82 6 10.1016/j.diabres.2008.09.012 18954918

[B8] Forbes JM Fukami K Cooper MF Diabetic nephropathy: where hemodynamics meets metabolism Exp Clin Endocrinol Diabetes 2007 115 69 84 10.1055/s-2007-949721 17318765

[B9] Kedziora - Kornatowska KZ Luciak M Blaszczyk Y Pawlak W Effect of aminoguadin on erythrocyte lipid peroxidation and activities of antioxidant enzymes in experimental diabetes Clin Chem Lab Med 1998 36 771 5 10.1515/CCLM.1998.137 9853804

[B10] Ha H Lee H Reactive oxygen species amplify glucose signalling in renal cells cultured under high glucose and in diabetic kidney Nephrology 2005 10 7 10 10.1111/j.1440-1797.2005.00448.x 16174288

[B11] Hernandez F Menendez S Wong R Decrease of blood cholesterol and stimulation of antioxidative response on cardiopathy patients treated with endovenous ozone therapy Free Rad Biol Med 1995 19 115 9 10.1016/0891-5849(94)00201-T 7635353

[B12] Rodriguez ZZ Guanche D Alvarez RG Rosales FH Alonso Y Schulz S Preconditioning with ozone/oxygen mixture induces reversion of some indicators of oxidative stress and prevents organic damage in rats with fecal peritonitis Inflamm Res 2009 in press 19274439 10.1007/s00011-009-0001-2

[B13] Coppola L Giunta R Verrazzo G Luongo C Sammartino A Vicario C Giugliano D Influence of ozone on haemoglobin oxygen affinity in type-2 diabetic patients with peripheral vascular disease: in vitro studies Diabetes Metab 1995 21 252 5 8529759

[B14] Chen H Xing B Liu X Zhang B Zhou J Zhu H Chen Z Ozone oxidative preconditioning protects the rat kidney from reperfusion injury: the role of nitric oxide J Surg Res 2008 149 287 95 10.1016/j.jss.2007.12.756 18262565

[B15] Barber E Menendez S Leon OS Barber MO Merino N Calunga JL Cruz E Bocci V Prevention of renal injury after induction of ozone tolerance in rats submitted to warm ischaemia Mediators Inflamm 1999 8 37 41 10.1080/09629359990702 10704088 PMC1781776

[B16] Unlucerci Y Bekpinar S Gurdol F Seferoglu G A study on the relationship between homocysteine and diabetic nephropathy in rats Pharmacol Res 2002 45 3 249 52 10.1006/phrs.2001.0942 11884223

[B17] Kedziora-Kornatowska KZ Luciak M Blaszczyk Y Pawlak W Effect of aminoguadin on erythrocyte lipid peroxidation and activities of antioxidant enzymes in experimental diabetes Clin Chem Lab Med 1998 36 771 5 10.1515/CCLM.1998.137 9853804

[B18] Knoll KE Pietrusz JL Liang M Tissue-specific transcriptome responses in rats with early streptozotocin-induced diabetes Physiol Genomics 2005 21 222 9 10.1152/physiolgenomics.00231.2004 15713786

[B19] Yechoor VK Patti ME Saccone R Kahn CR Coordinated patterns of gene expression for substrate and energy metabolism in skeletal muscle of diabetic mice Proc Natl Acad Sci USA 2002 99 10587 92 10.1073/pnas.142301999 12149437 PMC124982

[B20] Peralta C Xaus C Bartrons R Leon OS Gelpi E Rosello-Catafau J Effect of ozone treatment on ROS and adenosine production during hepatic ischemia-reperfusion Free Radic Res 2000 33 595 605 10.1080/10715760000301121 11200091

[B21] Wang C Chao L Chao J Direct gene delivery of human tissue kallikrein reduces blood pressure in SHR J Clin Invist 1995 95 1710 1716 10.1172/JCI117847 PMC295685 7535795

[B22] Ganong W Review of Medical Physiology 1999 19 London UK. Appelton and Lang Co 573 581

[B23] James W Martha L Mary A James M Margaret A Gali R Effects of Vanadate on renal hypertrophy and sorbitol accumulation in streptozotocin induced diabetes in rats Res Com-mun Pathol Pharmacol 1991 72 2 180 89 1876750

[B24] Bretaudiere JP Phung HT Baily M Direct enzymatic determination of urea in the plasma and urine with a centrifugal analyzer Clinical Chemistry 1976 22 1614 7 975505

[B25] Spencer K Analytical reviews in clinical biochemistry: the estimation of creatinine Ann Clin Biochem 1986 23 1 25 3532908 10.1177/000456328602300101

[B26] Trinder P Determination of blood glucose using 4-amino phenazone as oxygen acceptor J Clin Pathol 1969 22 2 246 10.1136/jcp.22.2.246-b 5776563 PMC474047

[B27] Nayak SS Pattabiraman TN A new colorimetric method for the estimation of glycosylated hemoglobin Clin Chem Acta 1981 109 267 74 10.1016/0009-8981(81)90312-0 7226519

[B28] Ohkawa H Ohishi N Yagi K Assay for lipid peroxides in animal tissues by thiobarbituric acid reaction Anal Biochem 1979 95 351 8 10.1016/0003-2697(79)90738-3 36810

[B29] Nishikimi M Rao NA Yagi K The occurrence of superoxide anion in the reaction of reduced phenazine methosulfate and molecular oxygen Biochem Biophys Res Commun 1972 46 849 54 10.1016/S0006-291X(72)80218-3 4400444

[B30] Kakkar P Das B Viswanathan PN A modified spectrophotometric assay of superoxide dismutase Indian J Biochem Biophys 1984 21 130 2 6490072

[B31] Bonaventura J Schroeder WA Fang S Human erythrocyte catalase: an improved method of isolation and revaluation of reported properties Arch Biochem Biophys 1972 150 606 17 10.1016/0003-9861(72)90080-X 5044042

[B32] Pagalia DE Valentine WN Studies on quantitative and qualitative characterization of erythrocyte GPx The Journal of Laboratory and Clinical Medicine 1967 70 158 6066618

[B33] Nishimura C Yamaoka T Mizutani M Yamashita K Akera T Tanimoto T Purification and characterization of the recombinant human aldose reductase expressed in baculovirus system Biochim Biophys Acta 1991 1078 171 8 1905957 10.1016/0167-4838(91)99006-e

[B34] Michael J Fowler MD Microvascular and Macrovascular Complications of Diabetes Clinical Diabetes 2008 26 77 82 10.2337/diaclin.26.2.77

[B35] Haidara M Yassin H Rateb M Ammar H Zorkani M Role of oxidative stress in development of cardiovascular complications in diabetes mellitus Current Vascular Pharmacology 2006 4 215 27 10.2174/157016106777698469 16842139

[B36] Abo-Salem OM El-Edel RH Harisa GE El-Halawany N Ghonaim MM Experimental diabetic nephropathy can be prevented by propolis: Effect on metabolic disturbances and renal oxidative parameters Pak J Pharm Sci 2009 22 2 205 10 19339234

[B37] Santo MS Santos DL Brain and liver mitochondria isolated from diabetic rats show different susceptibility to induced oxidative stress Diabet Metab Res Rev 2001 17 223 30 10.1002/dmrr.200 11424235

[B38] Cunningham JJ Micronutrients as nutriceutical interventions in diabetes mellitus J Am Coll Nutr 1998 17 7 12 9477383 10.1080/07315724.1998.10718729

[B39] Yang Y He X Chen S Wang L Li E Xu L Changes of serum and urine neutrophil gelatinase-associated lipocalin in type-2 diabetic patients with nephropathy: one year observational follow-up study Endocrine 2009 36 1 45 5 10.1007/s12020-009-9187-x 19390997

[B40] Toba H Sawai N Morishita M Murata S Yoshida M Nakashima K Morita Y Kobara M Nakata T Chronic treatment with recombinant human erythropoietin exerts renoprotective effects beyond hematopoiesis in streptozotocin-induced diabetic rat Eur J Pharmacol 2009 612 1-3 106 14 10.1016/j.ejphar.2009.03.065 19356735

[B41] Soto C Recoba R Barron H Alvarez C Favari L Silymarin increases antioxidant enzymes in alloxan-induced diabetes in rat pancreas Comp Biochem Physiol C Toxicol Pharmacol 2003 136 205 12 10.1016/S1532-0456(03)00214-X 14659454

[B42] Hassan WN Cantuti-Castelevetri I Denisova N Yee AS Joseph JA Paulson KE The nitrone spin trap PBN alters the cellular response to H_2_O_2_: Activation of the EGF receptor/ERK pathway Free Rad Biol Med 2002 32 551 61 10.1016/S0891-5849(02)00744-X 11958956

[B43] Al-Dalain SM Martínez G Candelario-Jalil E Ozone treatment reduces markers of oxidative and endothelial damage in an experimental diabetes model in rats Pharmacol Res 2001 44 391 6 10.1006/phrs.2001.0867 11712870

[B44] Candelario-Jalil E Al-Dalain SM León OS Oxidative preconditioning affords protection against carbon tetrachloride-induced glycogen depletion and oxidative stress in rats J Appl Toxicol 2001 21 297 301 10.1002/jat.758 11481663

[B45] Radko K William ES John FR Jessie NL Terry TO David CB Scott LM Sharon A Altered endothelial NO synthase targeting and conformation and Caveolin-1 expression in the diabetic kidney Diabetes 2006 55 1651 9 10.2337/db05-1595 16731827

[B46] Di Baccio D Castagna A Paoletti E Sebastiani L Ranieri A Could the differences in O_3 _sensitivity between two poplar clones be related to a difference in antioxidant defense and secondary metabolic response to O_3 _influx Tree Physiol 2008 28 12 1761 72 19193559 10.1093/treephys/28.12.1761

[B47] Martínez G Al-Dalain SM Menéndez S Giuliani A Alvarez H Fernández JI León OS Therapeutic efficacy of ozone in patients with diabetic foot Eur J Pharmacol 2005 31 151 61 10.1016/j.ejphar.2005.08.020 16198334

[B48] Nishikawa T Edelstein D Du XL Normalizing mitochondrial superoxide production blocks three pathways of hyperglycaemic damage Nature 2000 404 787 90 10.1038/35008121 10783895

[B49] Kaleem M Asif M Ahmed QU Bano B Antidiabetic and antioxidant activity of Annona squamosa extract in STZ-induced diabetic rats Singapore Med J 2006 47 670 8 16865205

[B50] Cardillo C Nambi SS Kilcovne CM Choucair WK Katz A Quon MJ Panza JA Insulin stimulates both endothelin and nitric oxide activity in the human forearm Circulation 1999 100 820 5 10458717 10.1161/01.cir.100.8.820

[B51] Azevedo-Martins AK Lortz S Lenzen S Curi R Eizirik DL Tiedge M Improvement of the mitochondrial antioxidant defense status prevents cytokine-induced nuclear factor-kappa B activation in insulin-producing cells Diabetes 2003 52 1 93 101 10.2337/diabetes.52.1.93 12502498

